# Transposition of the Great Arteries With Intact Ventricular Septum and Fetal-Onset Dilated Cardiomyopathy in a Neonate With a Homozygous NEXN Variant

**DOI:** 10.1155/crpe/7114473

**Published:** 2025-11-26

**Authors:** Daniel Geer, Emma Wakeling, Ewa Goljan, Hannah Robinson, Justine Swain, Craig Laurence

**Affiliations:** ^1^Department of Paediatric Cardiology, Great Ormond Street Hospital for Children NHS Foundation Trust, London, UK; ^2^North East Thames Regional Genetics Service, Great Ormond Street Hospital for Children NHS Foundation Trust, London, UK; ^3^Exeter Genomics Laboratory, Royal Devon University Healthcare NHS Foundation Trust, Exeter, UK; ^4^Department of Paediatrics, Mater Dei Hospital, Msida, Malta

**Keywords:** dilated cardiomyopathy, fetal-onset cardiomyopathy, NEXN variant, transposition of the great arteries

## Abstract

*NEXN* variants have previously been described as a cause of both pediatric- and adult-onset dilated cardiomyopathy but are not known to be associated with complex congenital heart disease. We report a case of an antenatal diagnosis of transposition of the great arteries with intact ventricular septum and fetal-onset dilated cardiomyopathy in a neonate with a loss-of-function homozygous frameshift variant in the *NEXN* gene, c.1589_1590del p.(Arg530Lysfs∗3). Both parents were found to be asymptomatic heterozygous carriers of the same variant at the time of diagnosis.

## 1. Introduction

Transposition of the great arteries (TGA) is a cyanotic congenital heart lesion characterized by discordant ventriculoarterial connection accounting for 3% of all congenital heart diseases and 20% of all cyanotic congenital heart lesions with an overall prevalence of 4.7 per 10,000 live births [1]. TGA has been described in association with heterotaxy syndromes and 22q11 deletion syndromes [2]. The genetic etiology of TGA remains poorly understood, although some ciliary genes have been implicated in the pathogenesis of TGA [3] and there is evidence suggestive of a polygenic inheritance pattern [4].

*NEXN* encodes the Z-disc protein nexilin, which has been shown to be a pivotal component of the junctional membrane complex in mice and plays an important role in the formation of T tubules [5]. *NEXN* variants have been shown to cause Z-disc instability in zebrafish models as well as in human tissue from myocardial biopsy [6]. Loss of nexilin function in a knock-out mouse model has been shown to cause a rapidly progressive cardiomyopathy in homozygous mice with left ventricular dilatation, wall thinning, and impaired cardiac function. In addition, there was evidence of collagen and elastin deposition mimicking endocardial fibroelastosis within the left-ventricular cavity by Day 6 of life. All these mice died by Day 8 of life [7].

In humans, *NEXN* variants have previously been shown to be associated with dilated cardiomyopathy (DCM), predominantly in adults with average age of onset above 50 years in the context of heterozygous variants [6].

In addition, a small number of patients have been reported with biallelic *NEXN* variants in association with pediatric-onset DCM. Fetal presentation has been reported in 9 cases from 6 families in association with compound heterozygous or homozygous loss-of-function variants [8–13] (Table 1). In most of the documented cases of biallelic *NEXN* variants, there was either *in utero* demise or death in the neonatal period. Additionally, a child was reported who presented at 8 weeks of age with severe neonatal-onset DCM who was found to have compound heterozygous missense *NEXN* variants [14].

Although *NEXN* variants have not previously been described in cases of complex congenital heart disease, such as TGA, gain-of-function *NEXN* variants resulting in GATA4 suppression have been described as the cause of secundum atrial septal defects in both mouse models and humans [15].

Additionally, *NEXN* variants have also recently been implicated as a possible etiological factor in coronary artery fistula formation in a child with a giant coronary artery aneurysm who also had a patent ductus arteriosus. However, this child also had a missense variant in *ABCC6,* NM_001171:c.1312G > A p.(Val438Met), in addition to a heterozygous truncated variant in the *NEXN* gene, NM_144573:c.298G > T p.(Gly100∗) [16].

We describe a case of antenatally diagnosed TGA with intact ventricular septum and abnormal coronary artery anatomy in conjunction with fetal-onset DCM in the context of a loss-of-function homozygous *NEXN* frameshift variant.

## 2. Case Report

The patient was the first baby of healthy, Maltese parents who were not known to be related. There was no family history of congenital heart disease, cardiomyopathy, or sudden death in other family members.

Following an antenatal diagnosis of TGA at the anomaly scan, the mother was referred to the fetal cardiology service. Fetal echocardiography confirmed the diagnosis of TGA with intact ventricular septum. However, there was additional evidence of fetal cardiomyopathy with cardiomegaly and moderate biventricular systolic dysfunction which persisted throughout the pregnancy.

The baby was born in poor condition following an elective cesarean section at 37 weeks' gestation requiring intubation and ventilation at 5 min of age due to profound hypoxia. Peripheral access was secured, and a prostaglandin infusion started as per the antenatal plan. Empirical broad-spectrum antibiotics were started.

The baby was reviewed by an interventional cardiology consultant at 1 h of age at which point the preductal saturations were ∼30% with postductal saturations of ∼50% and a heart rate of 50 bpm. Echocardiography showed an inadequate interatrial communication and impaired ventricular function. An emergency balloon atrial septostomy was performed in challenging conditions with ongoing hemodynamic instability. Following this, there was a marked improved in oxygen saturations to 72% (pre- and postductal), although the patient continued to intermittently desaturate and require inotropic support for hemodynamic instability that was likely due to the underlying myocardial dysfunction.

**Figure 1:**
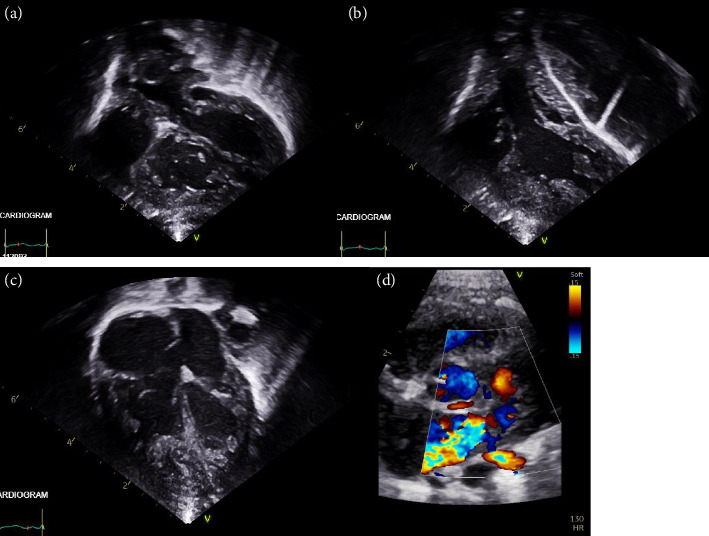
Postnatal echo: (a) subcostal view demonstrating pulmonary artery arising from the left ventricle, (b) subcostal view demonstrating aorta arising from the right ventricle, (c) apical 4-chamber view demonstrating large interatrial communication and hypertrabeculations of both ventricles, and (d) parasternal short-axis view demonstrating the circumflex artery running posteriorly to the pulmonary artery.

On Day 1 of life, the patient was transferred to the Cardiac Intensive Care Unit at Great Ormond Street Hospital. A detailed transthoracic echocardiogram confirmed the diagnosis of TGA with intact ventricular septum with an adequately sized interatrial communication following the previous balloon septostomy (Figure 1). Abnormal coronary anatomy was identified with a prominent coronary ostium arising from sinus 2, giving rise to the right coronary, left anterior descending, and left circumflex arteries. Left (subpulmonary) ventricular systolic function was moderately impaired with an ejection fraction of 40% despite inotropic support. There was no evidence of elevated left (subpulmonary) ventricular systolic pressure in the context of invasive ventilation and inhaled nitric oxide. Both ventricles had abnormal and hypertrabeculated myocardium. At admission, the N-terminal pro-B-type natriuretic peptide (NT-proBNP) level was 21,200 pg/mL. A biochemical screen for metabolic causes of cardiomyopathy was normal.

Despite the period of profound hypoxia and hemodynamic instability in the early postnatal period, no focal neurological abnormalities were identified on neurology review and an electroencephalogram (EEG) was normal for age. An MRI brain showed some areas of microhemorrhage with two areas of diffusion restriction in the septum pellucidum and the right corona radiata which were not felt to represent a contraindication to cardiac surgery.

**Figure 2:**
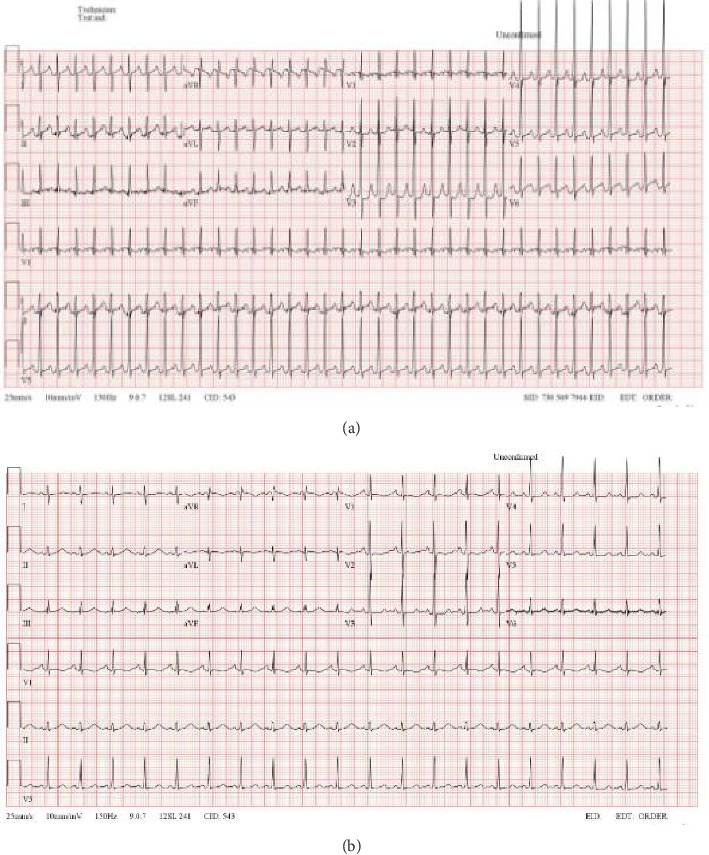
ECGs: (a) during episode of supraventricular tachycardia and (b) at baseline.

Over the first week, there was significant interval improvement in left (subpulmonary) ventricular systolic function and inotropic support was gradually reduced. The patient was successfully extubated to high-flow nasal cannula oxygen on Day 6, although low-dose adrenaline was required to maintain an adequate blood pressure. Serial echocardiograms showed significant improvement in ventricular function with the left (subpulmonary) ventricular ejection fraction improving to 57%. The patient had several episodes of supraventricular tachycardia (Figure 2(a)) with no apparent pre-excitation on the baseline ECG (Figure 2(b)), which were well controlled with propranolol.

Given the interval improvement in the baby's clinical status and ventricular function, a high-risk arterial switch operation with a LeCompte maneuver and partial closure of the interatrial communication was performed on Day 11. In theater, the ventricular function was subjectively felt to be impaired and impaired function was also noted on the postoperative echocardiogram despite good coronary artery flow. The chest was left open electively, and the patient was transferred back to the CICU. Of note, the patient had intermittent episodes of supraventricular tachycardia throughout the cardiac bypass run.

Postoperatively, the patient initially required significant hemodynamic support which was gradually reduced and stopped by postoperative day 13. The chest was closed on postoperative day 2, and the patient was extubated to high-flow nasal cannula on postoperative day 7. In view of ongoing ventricular dysfunction, the patient was established on captopril prior to discharge.

Gene-agnostic trio whole exome sequencing revealed a loss-of-function homozygous *NEXN* frameshift variant, NM_144573: c.1589_1590del p.(Arg530Lysfs∗3), which was classified as pathogenic according to the American College of Medical Genetics guidelines [17]. Both parents were shown to be heterozygous for the same variant, and the patient's father was found to have mildly impaired left ventricular function during a screening echocardiogram. Of note, no genetic abnormalities associated with an abnormal conduction system or arrhythmia were identified.

The patient has continued to make good clinical progress postoperatively. On clinical review at 5 weeks of age, he remained asymptomatic and well, although a repeat echocardiogram continued to demonstrate moderately impaired biventricular systolic function with a left ventricular ejection fraction of 41%. The patient has not had any further episodes of arrhythmia following the perioperative period and is currently off all anti-arrhythmic medications. This suggests that the supraventricular tachycardia seen in this patient in the pre/perioperative period is unlikely to be related to the underlying *NEXN* variant. Given the poor historical outcomes in children with biallelic *NEXN* variants and in fetal-onset NEXN-related DCM [8], the long-term prognosis for this patient remains guarded.

## 3. Discussion

Heterozygous *NEXN* variants are a well-recognized cause of later-onset cardiomyopathy. However, only a small number of patients have been reported to date with biallelic *NEXN* variants in association with severe, early-onset DCM, often presenting during the second or third trimesters of pregnancy.

We have described the first case of complex congenital heart disease in the context of a patient with a homozygous *NEXN* variant and early-onset DCM. Although gain-of-function *NEXN* variants have been implicated in the pathogenesis of atrial septal defects in mice and humans [15], no cases of complex congenital heart disease have been described in loss-of-function *NEXN* variants comparable to this case. Given the inverse relationship between NEXN expression and GATA4 expression described in mouse models [15], one could hypothesize that a loss-of-function *NEXN* variant may result in overexpression of GATA4. As an important cardiac transcription factor regulating cardiac development, overexpression of GATA4 may have a substantial impact on cardiac development resulting in congenital heart disease.

Although monoallelic gain-of-function *NEXN* variants have been associated with ASDs [15], genetic-related structural cardiac phenotypes can vary between individuals with variants in the same gene. It is feasible that the different, biallelic loss-of-function *NEXN* variant seen in this patient may be associated with other forms of congenital heart disease.

Based on the existing limited numbers of published biallelic *NEXN* variants, which have not been associated with complex congenital heart disease, it is not possible to definitively determine if the biallelic *NEXN* variant present in this patient played a causative role in the development of TGA or if the presence of TGA in this patient is merely coincidental. The majority of published *NEXN* variants are heterozygous, and it is not possible to exclude the possibility of a dosage effect in biallelic cases.

If additional reports of congenital heart disease in association with biallelic *NEXN* variants emerge with time, then further investigations into the potential mechanisms would be justified.

From a genetic testing standpoint, the increasing use of whole exome/genome sequencing in acutely unwell infants presenting with cardiomyopathy will help define the full spectrum of clinical severity associated with biallelic *NEXN* variants. *NEXN* should be included in next-generation sequencing gene panels for pediatric-onset cardiomyopathy, with both monoallelic and biallelic modes of inheritance considered. It is possible that initial reports have been biased toward more severely affected cases. Identification of further cases is needed to help advise parents of newly diagnosed infants regarding prognosis and management options.

Of note, in our case, there was no history of DCM in other family members prior to the diagnosis of this patient. In the family reported by Johansson et al. in 2021 [10], 2 out of 7 heterozygous carriers investigated following diagnosis were found to have DCM aged 37 and 59 years. This highlights the value of genetic testing in advising parents regarding their own need for cardiac screening for later-onset DCM. Diagnosis also provides reproductive options for future pregnancies and allows cascade screening to be offered to the wider family.

## Figures and Tables

**Table 1:** Reported cases of homozygous/compound heterozygous *NEXN* variants.

Author	Year	Variants (NM_144573)	Variant effect	No affected patients	Fetal/neonatal presentation	Outcome
Al-Hassnan et al. [8]	2013	Homozygous c.1582_1584del p.(Glu529del)	In-frame deletion	2	Neonatal (1 month)	
Bruyndonckx et al. [9]	2020	Homozygous c.1174C > T p.(Arg392∗)	Nonsense	1	Fetal	Neonatal death at 2 weeks of age
Sparks et al. [11]	2020	Compound heterozygous c.646C > T p.(Arg216∗) and c.1606_1607del p.(Lys536Valfs∗7)	Nonsense/frameshift	1	Fetal	Stillbirth
Rinaldi et al. [12]	2020	Compound heterozygous c.1756A > T p.(Lys586∗) and c.1909_1912del p.(Tyr637Alafs∗684)	Nonsense/frameshift	2	Fetal	Termination of pregnancy
Johansson et al. [10]	2021	Homozygous c.1302del p.(Ile435Serfs∗3)	Frameshift	3	Fetal	Intrauterine death at 24–30 weeks gestation
Thornton et al. [14]	2023	Compound heterozygous c.1955A > G p.(Tyr652Cys) and c.370G > A p.(Glu124Lys)	Missense/missense	1	Neonatal	Awaiting cardiac transplantation at 2 months
Picciolli et al. [13] (Patient 1)	2024	Homozygous c.1156dup (p.Met386fs)	Nonsense	1	Fetal	Clinically well at 24 months of age with LVEF 35%–40% on medical therapy
Picciolli et al. [13] (Patient 2)	2024	Homozygous c.1579_1584delp. (Glu527_Glu528del)	Frameshift	1	Fetal	Clinically well at 15 years of age with LVEF 40%–45% on medical therapy
